# EMRlog Method for Computer Security for Electronic Medical Records with Logic and Data Mining

**DOI:** 10.1155/2015/542016

**Published:** 2015-10-01

**Authors:** Sergio Mauricio Martínez Monterrubio, Juan Frausto Solis, Raúl Monroy Borja

**Affiliations:** ^1^Departamento de Ciencias Computacionales, Tecnológico de Monterrey, Campus Cuernavaca, Autopista del Sol Km 104, Colonia Real del Puente, 62790 Xochitepec, MOR, Mexico; ^2^Tecnológico Nacional de México, Instituto Tecnológico de Ciudad Madero, Avenida 1 de Mayo, Esquina Sor Juana Inés de la Cruz s/n, Colonia Los Mangos, 89440 Madero, TAMPS, Mexico; ^3^Tecnológico de Monterrey, Escuela de Ingeniería y Ciencias, Carretera Lago de Guadalupe, Km. 3.5, 52926, MEX, Mexico

## Abstract

The proper functioning of a hospital computer system is an arduous work for managers and staff. However, inconsistent policies are frequent and can produce enormous problems, such as stolen information, frequent failures, and loss of the entire or part of the hospital data. This paper presents a new method named EMRlog for computer security systems in hospitals. EMRlog is focused on two kinds of security policies: directive and implemented policies. Security policies are applied to computer systems that handle huge amounts of information such as databases, applications, and medical records. Firstly, a syntactic verification step is applied by using predicate logic. Then data mining techniques are used to detect which security policies have really been implemented by the computer systems staff. Subsequently, consistency is verified in both kinds of policies; in addition these subsets are contrasted and validated. This is performed by an automatic theorem prover. Thus, many kinds of vulnerabilities can be removed for achieving a safer computer system.

## 1. **Introduction**

In recent years computer security applications have grown significantly due to the increase of cybercrime. The international scientific community has been working in this field witnessing an increment in the number of publications on the subject. Failures of information systems due to a bad implementation of a security policy are usually explained in terms of policy misinterpretation or poor programming practices, including design problems [1].

Access control policies of an organization define the goals and constraints related to the actions of its members [2]. Within the study of computational security policies arise diverse complex challenges for which solutions are still pursued. To obtain accurate data for a system in a dynamic environment and to control the system are very complex tasks. An important part of the development software budget is devoted to critical applications, for testing them and ensuring they comply with the specifications. To guarantee that the policies built in the operational stage correspond with the policies defined in the planning stage is a very difficult issue. This is however paramount for health services, where monitoring the proper functioning of hospital computer systems in general requires an investment similar to the possible undesirable consequences.

**Figure 1:**
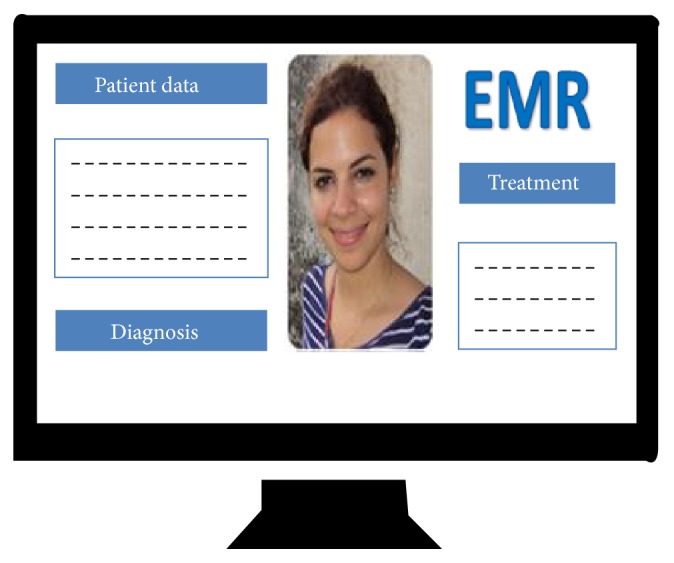
Example of electronic medical record.

The electronic medical record (EMR) contains the standard medical and clinical data gathered by physicians. EMR is a digital version of the paper chart, which contains all the medical history of patients and is used for diagnosis and treatment. Benefits of an EMR include the following: it is paperless, it tracks data over a long period of time, and it provides effective patient identification and preservation of records (vaccinations, blood pressure readings, X-rays). EMR information improves overall quality of care in a hospital practice. Nevertheless, the information stored in EMRs is not easily shared among physicians and administrative staff. The record of a patient may not have even been printed and mailed to specialists and other members of the care team; Figure 1 is an example of an EMR.

Confidentiality of an EMR is a main security issue. Thus, EMR is critical and extremely important for computer security in a hospital. Integrity, availability, and confidentiality of key software systems, databases, and data networks are major concerns throughout all sectors. Corruption, unauthorized disclosure, or theft of corporate resources could disrupt different tasks of a hospital and have immediate and serious financial, legal, safety, privacy, and public confidence impact [3].

One of the main motivations of this paper is based on the feasibility of defining and building an effective solution to verify the consistency of security policies once implemented. The problem is significantly more complex when there are an exceedingly large number of users in the system. This is due to a high number of operations that could cause inconvenient issues for the use and control of these medical systems.

A* directive policy*, referred to as M1, is a security policy, as defined by the executives of the hospital for which specific actions are to be carried out to guarantee security. By contrast, an* implemented policy*, referred to as M2, corresponds to the computing configurations of the system and all the performed actions for ensuring that all the M1 instructions are really implemented.

Formal methods, using logic as a tool, have been already used for policy verification [4–6]. They all have in common essentially these issues: (i) only directive policies are considered, and many operations can be required [7–9]; (ii) only consistency of implemented policies is verified, without proper contrast to directive policies. Perhaps the complexity of the problem has limited the work to solve the entire problem: consistency in every subset of policies and to contrast all of them to detect eventual contradictions. In fact the literature reports the next separated problems:

- Detection of policies implemented through logs for intrusion detection models [4, 8].
- General security models [10, 11].
- Logic models for security policy checking [6, 12].
- Implemented policy detection with data mining, [13].

In this paper, we introduce EMRlog, a model that contrasts directive versus implemented policies. Directive policies are written in a language defined for this purpose while the second ones are obtained after a process of data mining from log files. The paper is divided into four parts. The first is the introduction. In the second, a description of the two sets of policies is presented. The third part describes the extraction of the implemented policies by data mining. In the fourth section the verification EMRlog method is tested by simulation and experimentation. Finally, conclusions drawn from this study are presented.

## 2. Computer Security Policy

Computer security policies are defined as the measures taken to protect computers and their contents from unauthorized use. Computer security is the process of protecting applications, information, and computer resources. It often is taken as the provision of the next properties: (i) confidentiality, (ii) integrity, and (iii) availability [2]. The lack of security is a consequence of failures of one or more of these properties. When a hospital does not have a well-defined security policy, many problems can arise. In that case, information, hardware, and applications are left unprotected in face of many external and/or internal hacker attacks.

A security policy for access control describes how people may access documents or other computer resources. In order to obtain the correct information for security policies, access control should be described according to their environment in terms of three features [14]: subjects, objects, and actions.

The distinction between security policy and security properties is especially important [14]. The computer's version of a policy consists of a precise set of rules for determining authorization as a fundamental requirement for making access and control decisions. Authorization depends on the security attributes of users and information, unique IDs, and perhaps other information about the current state of the system [15].

Often a policy is a mixture of ad hoc rules for a certain period of time that could have evolved which can fall into an inconsistent enforced state. In addition, due to unclear, imprecise, or ambiguous policies the security of the systems can be reduced [14].

In addition, security policies of an organization define the objectives and constraints of all its elements: people, hardware, software, applications, and information. Basically, security policies are rules, which should be clearly written, completely understood, and correctly implemented. Therefore, the first step is to convince all the organization that these kinds of tasks must be performed. Moreover, an access control policy should describe the rights and obligations to be met by different types of users, as well as rules of conduct and how to make good use of the company resources. Some important points for developing security policies are as follows:

1. Identify security objectives.
2. Know to whom policies are addressed.
3. Configure security equipment adequately.
4. Contrast security policies systems of other hospitals and adopt good hospital security practices.

**Figure 2:**
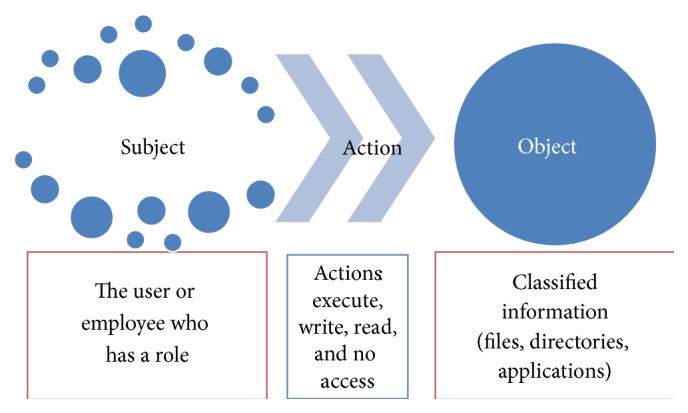
Interactions of subject-action-object [14].

Medical centers without security policy documentation can be in serious danger in regard to their EMR and medical attention processes. In addition, they can lose credibility in the quality of their service. By contrast, implementing security policies, an organization takes control of its operations and reduces the likelihood of vulnerabilities in the information to be exploited by an external or internal threat. As Figure 2 shows, the components of an access control policy are subject, objects, and actions. Subjects take certain roles, which enable them to perform some actions with restrictions about classified information according to their profiles.

To represent particular security policies on primary data files is required to find a scheme consisting of the main components involved in the management, distribution, and maintenance of information [16]:

*(i) Subjects*. They represent a dynamic entity within the organization and its employees (who occupy a hierarchical level according to their working activities) and the implementation of processes or applications that directly influence the status of an information file.

Examples of subjects in a hospital system are doctors, nurses, and administrative staff. These entities, through a specific access action, alter the structure or content of an information file, which must preserve its properties of integrity, confidentiality, availability, and authentication.

Subjects require a set of constraints, which, in turn, one should establish based on the roles that individuals play within the organization [12]. For each subject, the active role is the one that the subject is currently using [3]:

(1) $$\begin{array}{c} \text{AR}\left(\;s\text{: subject}\;\right)=\left\{\;\text{the active role for subject }s\;\right\}. \end{array}$$

Each subject may be authorized to perform one or more roles:

(2) $$\begin{array}{c} \text{RA}\left(\;s\text{: subject}\;\right)=\left\{\;\text{authorized roles for subject }s\;\right\}. \end{array}$$

Each role may be authorized to perform one or more transactions, in symbols: TA(*r*: role) = {transactions authorized for role *r*}. Subjects may execute transactions. Predicate exec(*s*, *t*) is true if subject *s* can execute transaction *t* at the current time; otherwise it is false.

*(ii) Actions*. They represent permissions for some subjects to realize some actions on some objects. Specific controls must be made by the system for any user action. Each organization specifies its own security rules. Some* roles* may have the permission, prohibition, or obligation to do some* activity* on some database. 

*(iii) Roles*. Three basic rules are required [3].

*(1) Role Assignment*. A subject can execute a transaction only if the subject has selected or been assigned a role:

(3) $$\begin{array}{c} \forall s\text{: subject,}\;\;t\text{: tran,}\;\;\left(\;\text{exec}\left(\;s,t\;\right)⟹\text{AR}\left(\;s\;\right)\neq ⌀\;\right). \end{array}$$

The identification and authentication process (e.g., login) is not considered a transaction. All other user activities on the system are conducted through transactions. Thus all active users are required to have some active role.

*(2) Role Authorization*. A subject's active role must be authorized for the subject:

(4) $$\begin{array}{c} \forall s\text{: subject},\;\left(\;\text{AR}\left(\;s\;\right)\subseteq \text{RA}\left(\;s\;\right)\;\right). \end{array}$$

Together with rule 1, this rule ensures that users can take on only roles for which they are authorized [3].

*(3) Transaction Authorization*. A subject can execute a transaction only if the transaction is authorized for the subjects active role:

(5) $$\begin{array}{c} \forall s\text{: subject},\;t\text{: tran},\;\left(\;\text{exec}\left(\;s,t\;\right)⟶t\in \text{TA}\left(\;\text{AR}\left(\;s\;\right)\;\right)\;\right). \end{array}$$

Together with rules 1 and 2, rule 3 ensures that users can execute only transactions for which they are authorized [3].

*(iv) Objects*. They represent information (the main resource of any organization), which must necessarily qualify for the implementation of access mechanisms that protect safety by restricting unauthorized users thus avoiding huge losses that impact the objectives and goals of the company. For example, the EMR database elements can take the role of objects.

**Figure 3:**
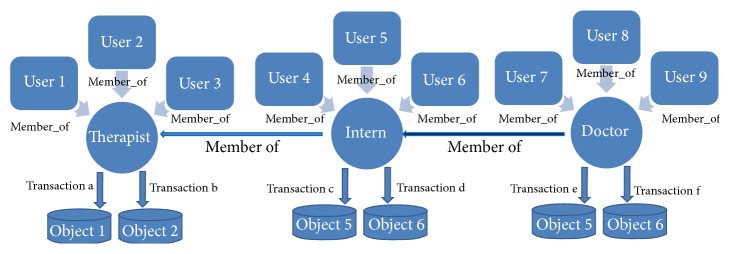
Multirole relationships.

A security policy is adequate if it helps in setting security goals including integrity, availability, confidentiality, authentication, and nonrepudiation, without hindering the mission of the organization for which they are developed. In [3] role based access control is explained via “the Therapist example,” where a healer can take on several roles (Figure 3): Therapist, Intern, and Doctor. In this case, the role Doctor implies access to all the transactions defined for the roles Intern and Therapist, as well as those of a Doctor. On the other hand, the Intern role implies only transactions of the Intern and Therapist and not those of a Doctor. Finally, the role Therapist only allows access to those resources defined for just a Therapist.

### 2.1. Policy Review Methods Using Predicate Logic

Logic is a tool that helps disambiguate natural language using a more precise, simple, and clear mathematical language. In EMRlog, natural language policies are transcribed into predicate logic. These security policies are mainly of either two types, permitting or denying. A permitting (resp., denying) security policy conveys the conditions under which someone, the subject, is allowed (resp., forbidden) to perform an action on some object. Accordingly, the vocabulary of our language is assumed to contain at least four collections of predicate relations. One denotes subjects (agents, processes, and officers), one denotes objects (files, directories, databases, and applications), one denotes actions (read, write, execute, and not access), and another denotes constraints (roles).

The predicate expression permitted(*S*, *A*) has two terms: *S* and *A*, identifying a subject and an action, respectively. The complete expression permitted(*S*, *A*) means that the subject *S* is allowed to carry out action *A*. Besides an action over some terms of type of actions can be described. Thus, in general a security policy is a sentence, which has the next form [12].

 Security policy standard form:
  (6) $$\begin{array}{c} \forall X_{1}\text{: }T_{1},\ldots ,X_{n}\text{: }T_{n},\;\left(\;C⟶\left[\;\neg \;\right]\text{permitted}\left(\;S,A\;\right)\;\right). \end{array}$$

The definition of literal comes from Latin litera/littera letter, alphabetic sign. In programming area, literal is a constant made available to a process, by inclusion in the executable text. Most modern systems do not allow texts to modify themselves during execution, so literals are indeed constant; their value is written at compile-time and is read-only at run time. In (6), *C* is a conjunction of literals *S* and *A* which are the terms previously defined, and [¬]permitted(*S*, *A*) indicates that *S* does not have permission of action *A*; in contrast, when the negation sign is not present in (6), this predicate declares that subject *S* does really have permission of execute action *A*.

A policy in the form of (6) is in the standard policy form [8]. Standard policies are generally enough to express any security policies (The work in [12]). For example, let us consider the next examples of policies taken from [12]:

1. Security officers may edit the password file.
2. Anyone who is allowed to edit a file may read it.
3. Anyone who is forbidden from reading a file may not edit it.
4. Employees may read all the information associated with their department of affiliation.

These four policies can be expressed as follows [12].

 Security policies example:
  (7) ∀X:  staff.  postX,officer,sec⟶permittedX,writepasswords,
  (8) ∀X:  staff.  ¬postX,officer,sec⟶¬permittedX,writepasswords,
  (9) ∀X:  staff.  F:  info.  permittedX,writeF⟶permittedX,readF,
  (10) ∀X:  staff.  F:  info.  ¬permittedX,readF⟶¬permittedX,writeF,
  (11) ∀X:  staff.  Y:  post,  Z:  dpt,  F:  info.  postsX,Y,Z∧∈F,Z⟶permittedX,readF.

In this sentence, post(*X*, officer, sec) means “*X* is an officer to the security department” and permitted(*X*, write(password)) means “*X* is allowed to write object* password*, taken to be a file.”

Once translated from natural language to predicate logic, the next step is to validate consistency using a theorem prover. An important good practice adopted in EMRlog is rule ordering. General rules appear first, followed by specific rules.

### 2.2. Using Data Mining for Implemented Policies (M2)

Implemented policies, M2, are policies as configured in the systems. In the literature, only security policies are checked for consistency using formal verification. However, once these policies are implemented some errors like the following can arise:

1. Policy misinterpretation/misunderstanding.
2. Policy omission, either by mistake or by intension.
3. Policy inconsistency, for example, giving more privileges to users.

**Figure 4:**
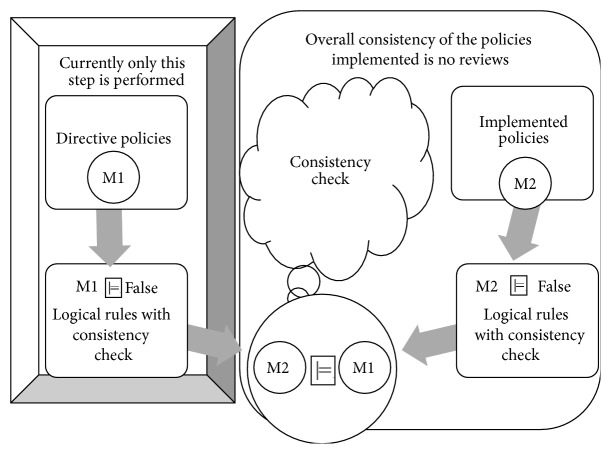
Directives and implemented policies verification.

Data mining is usually defined as the task of identifying patterns of interest and describing them concisely and meaningfully or as the automatic extraction of implicit interesting patterns in large data collections. As discussed before, in our approach we consider two universes of policies. One, named M1, is the subset of directive policies, which has been shown consistent through an axiomatic logic process. The other, named M2 (see the right hand side of Figure 4), represents the implemented policies. M2 is obtained using a data mining technique, actually, one that is very common, namely, decision trees. In the center of Figure 4 the bubble “consistency check” shows the consistency task that should be done between M1 and M2. More precisely, consistency of M1 with respect to M2 is represented by M2 *⊨* M1. A very well known method is resolution, which usually works by contradiction, negating the statement to be verified. The main impact of this paper is to verify whether policy directives are actually implemented in the sites of the organization.

### 2.3. System Roles

Table 1 shows the types of roles that are commonly found in every system. The classic nomenclature for permissions is used for fast and efficient understanding; for instance, RWX for a manager means that this role (i.e., manager) has permission: read, write, and execute; another role cannot access the system. To keep this nomenclature is very important because it represents the basis for interpretation for building any secure computer system [14].

### 2.4. EMRlog Method Steps

**Figure 5:**
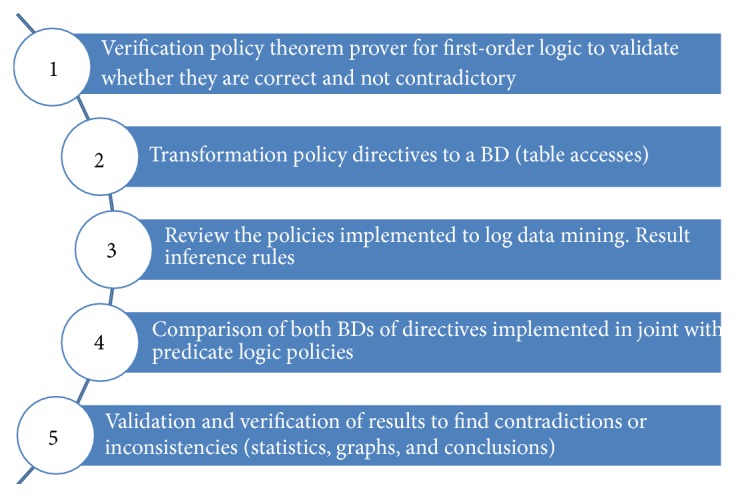
EMRlog methodology steps.

The EMRlog methodology is based on five steps as shown in Figure 5.

The consistency verification of directive and implemented policies (M1 and M2) for the EMR is made by the EMRlog method, which performs the verification of both M1 and M2 (Figure 7). The explanation of the five steps of EMRlog method is as follows:

- The first step is the verification of the directive policies set. This is done by using a theorem prover for predicate logic which by a refutation method checks if this set is inconsistent or not. In this paper Prover9 is used to validate the directive policies M2.
- The second step is the transformation of the directive policies into a database. The method classifies the three entities previously explained (subjects, actions, and objects).
- The third step is to review the policies implemented in the EMR data base log (including the log of the system). This database is analyzed using the C4.5 algorithm [17]. The inference rules obtained by this algorithm are used to review the policies implemented in the system. The mined rules are translated into predicate logic for further verification of correctness.
- The fourth step is the comparison of both databases obtained in the second step (i.e., the directive and implemented policies). For this task the automatic theorem prover is used. As a result, the entire set of policies (M1 and M2) is checked. This process is also illustrated in Figure 4.
- Finally, using the results of the previous step, the fifth step is the interpretation of the report of the automatic theorem prover. The possible results are as follows:
  - M1 policy directives are not implemented in M2.
  - The policies implemented in M2 contradict the M1 policies.
  - There are policies in M2, which do not have equivalent policies in M1.
  - The M1 policies directives are correct and consistent with policies implemented in M2. Thus, M1 *⊨* M2. In this case, EMR security is consistent, or M2 follows the policy directives.

### 2.5. EMRlog Implementation

Figure 4 shows that the directives policies M1 are contrasted with the implemented policies M2. Security policies are formalized in syntax of Prover9 in order to validate their consistency. This section outlines the implementation of the five steps of EMRlog through some examples.

**Step 1 (M1 directive policies verification).**

To continue our example, we use the same directive policies and then translate into a Prover9 (see policies (1)–(4) listed in Section 2.1).

Policies (1) to (4) are in predicate logic (see (7)–(11)).

Next, policies (7) to (11) are translated into Prover9 for validation:

(a) *set(auto).*
(b) *formula_list(usable).*
(c) *(all x (staff (x) *→* (post (x), officer_sec) *→* permitted(x, write (passw)))).*
(d) *(all x (- staff (x) *→* -(post (x), officer_sec) *→* -permitted(x, write (passw)))).*
(e) *(all x (staff (x) *→* all F info(F) *→* (permitted (x,write(F) *→* permitted (x,read(F)))))).*
(f) *(all x (staff (x) *→* F (info(F)*→* permitted(x,read(F) *∧*- (permitted(X,write(F))))))).*
(g) * (all x all y all z all f (staff(x) *→* (pos(y) *→* (dept(z) *→* (info(f) *→* (post(x, y, z) & belongs2(f, z) *→* perm(x, read(f)))))))).*

Instructions (a) and (b) are specific to Prover9; (c) to (g) are the predicate formulas. For this step, we adopted the approach reported in [12].

**Step 2 (M1 directive policies transformation).**

Once the validation is correct and rules are found not to be contradictory, the next step is to send the directive policies in a database that has the attributes of Table 2.

**Step 3 (M2 implemented policies review).**

Firstly, the M2 implemented policies are extracted from the EMR database log using C4.5 algorithm. Every SQL Server database has a transaction log that records all transactions and the database modifications made by each transaction. The benefit by the transaction log is the recovery of individual transactions. EMRlog method extracts this information from the EMR database log using C4.5 algorithm. This program generates a classifier in the form of a decision tree used to classify the user-object-action at the root of the tree and moving through until a leaf is encountered. When this process finally leads to a leaf, the class of the user-object-action is predicted. Finally, these results are translated manually to policies from natural language into predicate logic. In the example the implemented policies M2 found are the same as the directive policies M1. For instance, see policies (1)–(4) listed in Section 2.1.

Policies (7) to (11) are manually converted in predicate logic as shown in Step 1. Next M2 is translated into Prover9 theorem software for validation as shown in Step 1.

M2 implemented policies results are as follows:

 ≫ ACCESS RESULTS. The access and actions detected are written in Table 3.

**Step 4 (M1 and M2 comparison).**

The next step is to compare inconsistencies via both databases (i.e., M1 and M2 databases).

**Step 5 (M1 and M2 validation).**

Both worlds M1 (directive policies) and M2 (implemented policies) are used to make comparisons. Interpretation of results: if the same results are found in both worlds (M1 and M2), then both worlds are correct. Otherwise, there are some contradictions or inconsistencies which can be detected by using axiomatic test detection software.

## 3. EMRlog Testing

Because of the multiple interactions of the users with the system, testing becomes a very complex problem and a real challenge in a complete environment. The EMRlog was tested with data from an international company that has 1000 directive policies, which should be implemented in five different sites. In this case, the hospital has around 18,000 users and each user has different types of access in the system. There are five different sites:

 Site 1 = hardware (laptop, desktop).
 Site 2 = operating system (Windows, OS).
 Site 3 = network (TCP/IP).
 Site 4 = data base (Oracle, Informix).
 Site 5 = applications (EMR, ERP).

Each site has different privileges for each user (see Table 4).

Table 4 shows the names and the user access type. However, in a real environment, a user does not have a single access site. The number of interactions of access types for every user is different for each organization. For example, in the present case, the hospital has 18,000 users and at least five sites for each user, and the number of interactions will be 90,000. On the other hand, the systems are dynamic and always changing and the basic actions of users (create, update, modify, and delete) can be constantly applied. As a consequence, the number of total interactions can be extremely large and the problem becomes too difficult.

The number of access types for each user is already defined as follows: manager, designer, writer, reader, and no access. These access types are very common in the security area.

## 4. Data Mining for EMRlog File Information Extraction

Data mining is the stage forming part of the process known as knowledge discovery in databases (Databases on Knowledge Discovery, KDD) which provides a number of automated tool advantages to analyze data to find ways to increase efficiency in an organization and information sharing. The KDD is defined as a task for identifying pattern [13, 18] which presents a useful method of classification for intrusion detection systems; these methods are general strategies for intrusion detection. There are two categories under this misuse detection classification and anomaly detection. Data mining refers to a process of nontrivial extraction of implicit previously unknown and potentially useful information from databases to discover patterns of intrusions. It then applies a metalearning classifier to learn the signature of attacks. Data mining attempts to extract implicit previously unknown and potentially useful information from data. Applications of data mining to anomaly detection include ADAM (audit data analysis and mining) [13], IDDM (intrusion detection using data mining) [19], and eBayes [20].

Data mining has a set of techniques and technologies to explore large databases, automatically or semiautomatically, with the aim of finding repetitive patterns, trends, or rules that explain the behavior of the data in a given context [21]. Specifically, data mining performs a specific task with the data that have passed through the stages of cleaning and sorting:

- Exploratory data analysis.
- Descriptive modeling.
- Predictive modeling: classification and regression.
- Discovery of patterns and rules.
- Content recovery.

C4.5 algorithm is for policies implemented through data mining decision tree C4.5. After cleaning data, the next step used in data mining is leaving only the relevant data [18]; for this analysis ID3 and C4.5 are very popular algorithms to find patterns in the database [17]. In a hospital case these patterns are useful for identifying the actions really taken for the different roles of the users of the system.

The decision trees use (A) an entropy measure for classifying discrete values in several branches of the tree; (B) a training set and a test set; (C) the way to make partitions guided by the information gain and gain ratios heuristics. This paper uses C4.5 for the EMRlog to find the following:

1. User privileges consistent with what directive policies require.
2. Report of users active or inactive in the system.
3. Detection of abnormal situations such as the following:
  - Users with more privileges than the directives policies force.
  - Users that no longer work for the company and yet continue using the system.
  - Users that have restricted access to confidential data bases yet they can access.

**Figure 6:**
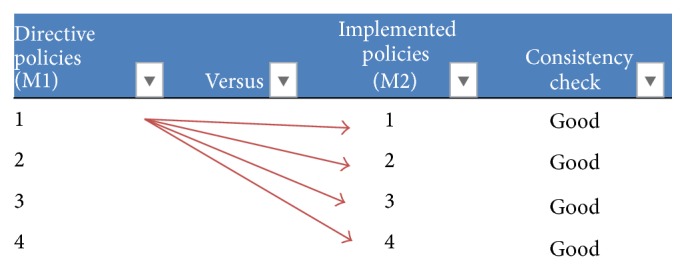
Consistency check M1 versus M2.

**Figure 7:**
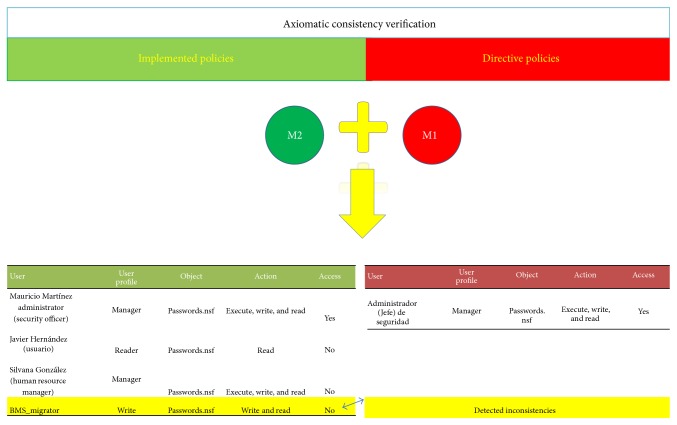
EMRlog consistencies verification.

**Figure 8:**
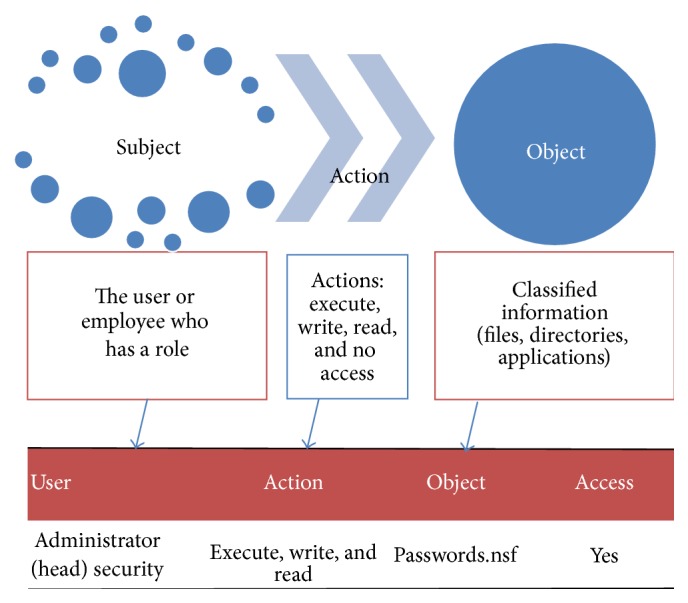
Transformation policies into access tables.

The basic methodology for constructing regression trees is the one used in decision trees [17]. When the decision tree was built, then policies implemented M2 are transformed into an access table in order to work as shown in Figures 6 and 8 with the directive policies M1, which are contrasted with implemented policies M2 in order to review the consistency (as is shown in Figure 6).

**Figure 9:**
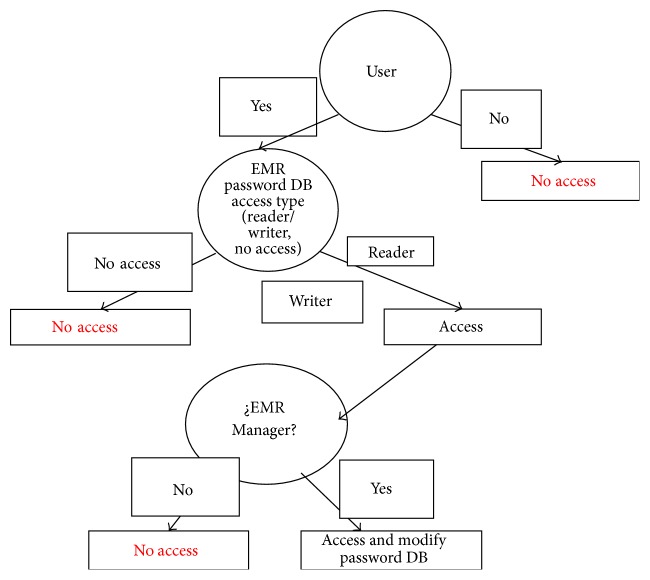
EMR decision tree.

**Figure 10:**
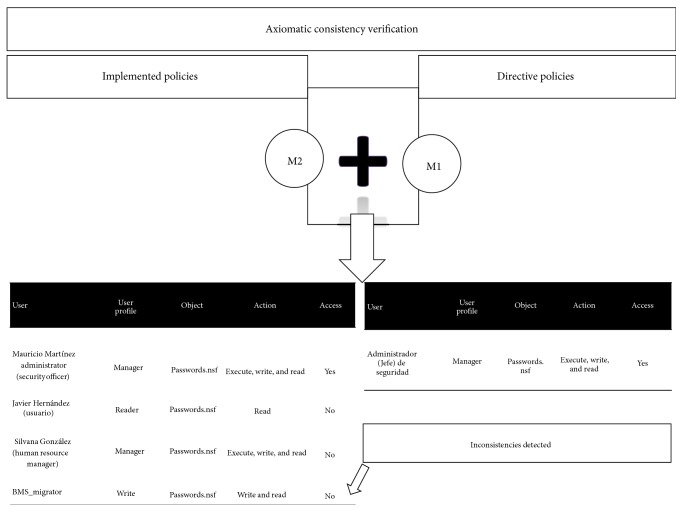
Consistency verification.

**Figure 11:**
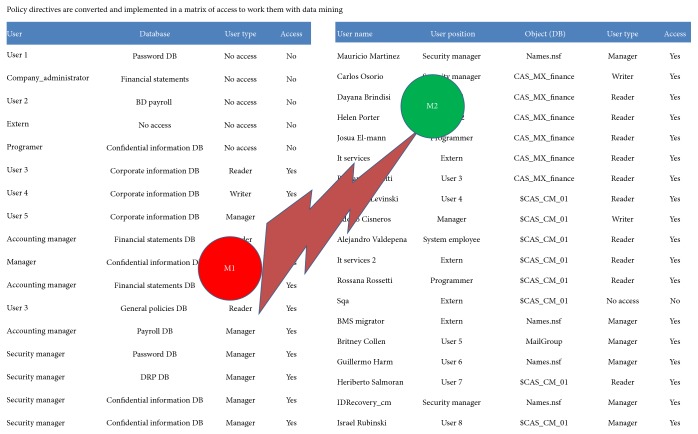
Tables M1 and M2 contrasted.

In Figure 9 the policies are reviewed by decision trees; Weka software helps to identify the policies poorly designed and also for finding deviations in directive policies. Finally, in Figures 10 and 11 a comparison of the two universes of discourse is done for detecting inconsistencies.

Figure 11 shows two tables with data of the M1 directive policies and M2 implemented policies. The two tables are contrasted in order to find contradictions or inconsistencies.

## 5. Results

As part of the validation process, general security policies of the two examples shown in previous sections were deliberately denied once the document presents no inconsistencies. However, the result produced by a logic theorem prover does not reflect inconsistencies. This is because the general security policies represent goals that meet the particular policies specified. If a particular policy contradicts any safety goal, the demonstrator finds inconsistencies. The process of forming correct security policies is the first step of this paper. Though the time used in the capture and validation of security policies is relatively large, it does not exceed a larger period of time than that used in the description of policies. If directives policies are validated and no contradiction was found in them, directives company policies are correct and the process continues to Step 2. Based on this research five incorrect cases were defined on purpose. Then the directives policies M1 and implemented policies M2 were tested. The first case has a fictitious role of manager (i.e., the BMS_Migrator). In this case the user should not have access to the system. In the second case, two users have the profile reader, and there was no policy directive M1 to issue that order, and M2 has more security policies. Finally, in the third case, two policies are violated because the writer profile has a BMS_Migrator; as a consequence, the user should not have access to the entire system but has access (incorrectly) as a manager. The summary of these policies is as follows:

- M1 policy directives are not implemented in the M2 sites.
- M1 policies contradict policies and the policies implemented in the M2 sites.
- There are more policies in M2 than that defined in M1.
- The implemented policies M2 are correct and consistent with the directive policies M1. Thus, M2 *⊨*= M1.

In test cases where intentionally designed policies are not consistent with the policies implemented effectively the following causes are found:

1. M1 policies are not completely implemented in M2.
2. M1 and M2 are contradictory.
3. There are policies in M2 which are not defined in M1.

**Figure 12:**
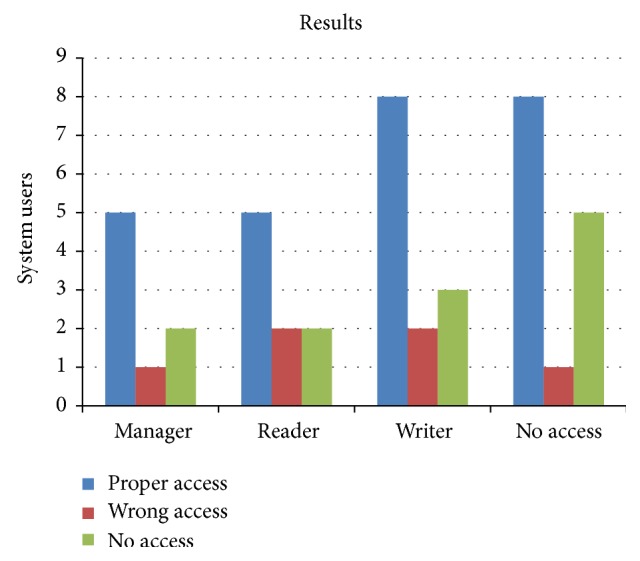
Results graph from the policies comparison.

The latter cases were tested and the results are shown in Figure 12. The interpretations derived from these results are the following:

1. The EMRlog method is effective.
2. Effectiveness was measured with tests that simulated differences between the policies implemented.
3. As a result of our tests it was found that policies are consistent in 99% of cases applied.

## 6. Conclusion

The EMRlog method is innovative because currently the security systems ignored validating both worlds, due to the influence of computer security. The EMRlog method checks the following:

- The EMRlog method verifies EMR inconsistencies between implemented and directive policies.
- The EMRlog method improves the security. The users as physicians, nurses, and administrative staff only see records of their patients and others are allowed to see.
- Only authorized users will be able to see the medical records assigned to them.

Finally, the proposed method is able to determine if a set of policies is valid. EMRlog contrasts directive and implemented policies using an automatic theorem prover, showing if a set of security policies are consistent. The definition and verification of integrity policies in EMRlog were successfully implemented for a case study.

## Figures and Tables

**Table 1:** Types of roles in the EMRlog system.

Role	Description	Nomenclature
Manager	Administrators have the privilege to create users, modify databases, make backup, copy, and so forth. Read, write, and execute.	RWX

Writer	Users can read or write to a database.	RW

Reader	Users who can only read information. Ex. E-mails corporate. Read.	R

No access	Users without access to the system. No access.	NA

**Table 2:** Types of roles in the EMRlog system.

User	User profile	Object	Action	Access
Administrator (security officer)	Manager	Passwords.nsf	Execute, write, and read	Yes

**Table 3:** Types of roles in the EMRlog system.

User	User profile	Object	Action	Access
Mauricio Martínez administrator (security officer)	Manager	Passwords.nsf	Execute, read, and write	Yes

Javier Hernández (user)	Reader	Passwords.nsf	Read	No

Silvana González (human resource manager)	Manager	Passwords.nsf	Execute, read, and write	No

BMS_Migrator	Write	Passwords.nsf	Write and read	No

**Table 4:** Site names and user access type.

#	Site name	Type	User access type
1	Hardware	Laptop, desktop	Manager

2	Operating system	Windows 8.0 OS	Manager

3	Network	TCP/IP	Reader

4	Data base	Oracle database ver. 8i	Writer

5	Applications	SAP EMR ver. 4.5	Reader

## References

[1] Sapolsky H., Gholz E. (2014). *US Defense Politics: The Origins of Security Policy*.

[2] Askarov A., Myers A. (2011). Attacker control and impact for confidentiality and integrity. *Logical Methods in Computer Science*.

[3] Ferraiolo D. F., Kuhn D. R. Role-based access controls.

[4] Shankar N. (2009). Automated deduction for verification. *ACM Computing Surveys*.

[5] Schneider F. B. (2000). Enforceable security policies. *ACM Transactions on Information and System Security*.

[6] Wahsheh L. A., de Leon D. C., Alves-Foss J. (2008). Formal verification and visualization of security policies. *Journal of Computers*.

[7] van der Meyden R. (1996). The dynamic logic of permission. *Journal of Logic and Computation*.

[8] Halpern J. Y., Weissman V. (2008). Using first-order logic to reason about policies. *ACM Transactions on Information and System Security*.

[9] Cuppens-Boulahia N., Cuppens F., Cuppens-Boulahia N., Cuppens F. (2008). Specifying intrusion detection and reaction policies: an application of deontic logic. *Deontic Logic in Computer Science*.

[10] Bell D., LaPadula L. (1975). Secure computer systems: unified exposition and multics interpretation.

[11] Cuppens F., Cuppens-Boulahia N., Ghorbel M. B. (2007). High level conflict management strategies in advanced access control models. *Electronic Notes in Theoretical Computer Science*.

[12] Álvarez A., García K., Monroy R., Trejo L., Vázquez J., Álvarez A. V., García K. A., Monroy R., Trejo L. A., Vázquez J. (2006). A tool for managing security policies in organisations. *Advances in Information and Computer Security*.

[13] Noel S., Wijesekera D., Youman C., Barbara D., Jajodia S. (2002). Modern intrusion detection, data mining, and degrees of attack guilt. *Applications of Data Mining in Computer Security*.

[14] Gasser M. (1988). *Building a Secure Computer System*.

[15] Salerno R., Gaudioso J., Brodsky B. (2007). *Laboratory Biosecurity Handbook*.

[16] Vallabhaneni R. (2013). *Wiley CIA Exam Review Focus Notes 2013: Internal Audit Knowledge Elements*.

[17] Quinlan J. (1993). *C4.5: Programs for Machine Learning*.

[18] Fayyad U., Piatetsky-Shapiro G., Smyth P. (1996). From data mining to knowledge discovery in databases. *AI Magazine*.

[19] Abraham T. (2001). IDDM: intrusion detection using data mining techniques.

[20] Valdes A., Skinner K. (2000). Adaptive, model-based monitoring for cyber attack detection. *Recent Advances in Intrusion Detection*.

[21] Abdul Jaleel J., Sibi S., Aswin R. (2012). Artificial neural network based detection for instance, skin cancer. *International Journal of Advanced Research in Electrical, Electronics and Instrumentation Engineering*.

